# Evaluating the Applications of Health Information Technologies in China During the Past 11 Years: Consecutive Survey Data Analysis

**DOI:** 10.2196/17006

**Published:** 2020-02-10

**Authors:** Jun Liang, Ying Li, Zhongan Zhang, Dongxia Shen, Jie Xu, Gang Yu, Siqi Dai, Fangmin Ge, Jianbo Lei

**Affiliations:** 1 IT Center, Second Affiliated Hospital School of Medicine Zhejiang University Hangzhou China; 2 Department of Burn and Plastic Surgery Affiliated Hospital of Southwest Medical University Luzhou China; 3 Performance Management Department Qingdao Central Hospital Qingdao China; 4 Editorial Department of Journal of Practical Oncology, Second Affiliated Hospital School of Medicine Zhejiang University Hangzhou China; 5 IT Center, Children’s Hospital School of Medicine Zhejiang University Hangzhou China; 6 School of Medicine Zhejiang University Hangzhou China; 7 International Network Medical Center, Second Affiliated Hospital School of Medicine Zhejiang University Hangzhou China; 8 Center for Medical Informatics Peking University Beijing China; 9 Institute of Medical Technology Health Science Center Peking University Beijing China; 10 School of Medical Informatics and Engineering Southwest Medical University Luzhou China

**Keywords:** medical informatics, health information technologies, China, health care reform, hospitals

## Abstract

**Background:**

To achieve universal access to medical resources, China introduced its second health care reform in 2010, with health information technologies (HIT) as an important technical support point.

**Objective:**

This study is the first attempt to explore the unique contributions and characteristics of HIT development in Chinese hospitals from the three major aspects of hospital HIT—human resources, funding, and materials—in an all-around, multi-angled, and time-longitudinal manner, so as to serve as a reference for decision makers in China and the rest of the world when formulating HIT development strategies.

**Methods:**

A longitudinal research method is used to analyze the results of the CHIMA Annual Survey of Hospital Information System in China carried out by a Chinese national industrial association, CHIMA, from 2007 to 2018. The development characteristics of human resources, funding, and materials of HIT in China for the past 12 years are summarized. The Bass model is used to fit and predict the popularization trend of EMR in Chinese hospitals from 2007 to 2020.

**Results:**

From 2007 to 2018, the CHIMA Annual Survey interviewed 10,954 hospital CIOs across 32 administrative regions in Mainland China. Compared with 2007, as of 2018, in terms of human resources, the average full time equivalent (FTE) count in each hospital’s IT center is still lower than the average level of US counterparts in 2014 (9.66 FTEs vs. 34 FTEs). The proportion of CIOs with a master’s degree or above was 25.61%, showing an increase of 18.51%, among which those with computer-related backgrounds accounted for 64.75%, however, those with a medical informatics background only accounted for 3.67%. In terms of funding, the sampled hospitals’ annual HIT investment increased from ¥957,700 (US $136,874) to ¥6.376 million (US $911,261), and the average investment per bed increased from ¥4,600 (US $658) to ¥8,100 (US $1158). In terms of information system construction, as of 2018, the average EMR implementation rate of the sampled hospitals exceeded the average level of their US counterparts in 2015 and their German counterparts in 2017 (85.26% vs. 83.8% vs. 68.4%, respectively). The results of the Bass prediction model show that Chinese hospitals will likely reach an adoption rate of 91.4% by 2020 (*R*^2^=0.95).

**Conclusions:**

In more than 10 years, based on this top-down approach, China’s medical care industry has accepted government instructions and implemented the unified model planned by administrative intervention. With only about one-fifth of the required funding, and about one-fourth of the required human resources per hospital as compared to the US HITECH project, China’s EMR coverage in 2018 exceeded the average level of its US counterparts in 2015 and German counterparts in 2017. This experience deserves further study and analysis by other countries.

## Introduction

Health information technologies (HIT) can effectively improve the quality and efficiency of medical services, distribution of health care resources, safety in health care, and output of scientific research. Therefore, governments of various countries have set up ambitious plans to develop HIT and invested enormous amounts of money in this development, using HIT as an important starting point for the reformation of medical services and medical systems.

The US government invested $787 billion in the American Recovery and Reinvestment Act of 2009. In particular, $19 billion of this investment was used to promote nationalized and interoperable health information systems and implement them through the Health Information Technology for Economic and Clinical Health (HITECH) Act [1]. Its core Meaningful Use strategy has achieved initial results [2]. As of 2014, approximately 75.5% of US hospitals had at least a basic system with a defined set of functions applied in at least one hospital unit. About 69% of these hospitals supported the exchange of laboratory examination results, 65% supported exchange of radiological examination reports, 64% supported exchange of progress notes, and 55% supported exchange of medication histories, compared with 35%, 37%, 25%, and 21%, respectively, in 2008 [3]. The United Kingdom launched the National Programme for IT in 2005. By 2011, the utilization rate of electronic health records (EHR) for primary care was close to 100% [4], and the successful experience of the US HITECH Act was further introduced in 2014 [4].

China has been no exception to this trend. As early as the beginning of the second health care reform in 2010, the government adopted HIT as one of the “four beams and eight pillars” supporting health care reform [5] and successively promulgated 31 national policies and 134 technical standards covering all aspects of hospital, population health, and medical security system digitalization.

In order to build the HIT system, as detailed in the Healthy China 2020: Strategic Research Report released by the National Health Commission of the People’s Republic of China in 2012 [6], a national budget of US $10 billion will be invested to build the National Electronic Health Information System Project by 2020, more than one-seventh of the total investment of US $68 billion designated for the plan. As of 2015, the central government had actually invested more than US $3.5 billion. For details of the investment and expected results, see Multimedia Appendix 1. According to the latest administrative directive issued by the National Health Commission of the People’s Republic of China in August 2018, the use of electronic medical records (EMR) in hospitals should be included in the index system for hospital performance evaluation [7].

Despite the formulation of very active macro policies and the investment of a large amount of funds, governments of various countries have always faced significant challenges in the technological research and development, project implementation, effect evaluation, and speed of advancement of HIT. Governments, academic circles, and industries have constantly presented the relevant experience and lessons. Kruse et al [8] collected 3636 articles and selected 37 articles for final research; they found that 81% of the research projects believed that the HIT projects already implemented had a positive effect on the quality and cost of medical care. Gold et al [3] advanced the claim that although HITECH provides administrative and economic resources for the standards and interoperability of EHRs and HIT, the law does not stipulate how to achieve them. The US administrative system retains considerable autonomy for the private sector, making it even more difficult to reach a consensus under the current situation of relatively independent public power at the federal and state levels. This has led to a substantial delay in the implementation of HITECH. At present, it is too early to evaluate the final effect of HIT projects implemented between 2009 and 2015. Adler-Milstein et al [9] found that with the stimulation of HITECH, as of 2013, EHRs have been used in more than 50% of hospitals, with some regional differences; rural and small specialized hospitals lag far behind, potentially leading to problems of medical resource allocation.

As the largest country in the world in terms of population and number of hospitals and the second largest in total economic volume, China currently lacks relevant research on the application status, characteristics, and challenges of HIT in its hospitals. In this study, we try to answer the following questions:

- How can we describe, evaluate, and summarize the achievements and problems in China’s HIT development from 2007 to 2018?
- During this period, compared with countries with advanced HIT such as the United States, what are China’s characteristics in terms of the number and quality of HIT employees, capital and resource investment, network support environment, and application of clinical information systems (CIS) such as EMR?

## Methods

### Data Resources

Our data are from the 2007-2018 China Hospital Information Management Association (CHIMA) annual survey hospital information systems, which is the only national HIT industry survey covering a period of more than 10 years in China. Over the last decade or so, CHIMA [10] has used the questionnaire issued by the journal Chinese Digital Medicine to conduct continuous research on China's HIT application market in March of every year. The research area covered 34 administrative regions of mainland China. The institutions reviewed included general hospitals, specialized hospitals, traditional Chinese medicine hospitals, and integrated traditional Chinese and western medicine hospitals. The interviewees were chief information officers (CIOs) who were responsible for the information technology (IT) departments of the hospitals. The research method was designed with reference to the Healthcare Information and Management Systems Society (HIMSS) annual survey in the United States, and hospitals that did not respond in time received email and telephone notifications.

The CHIMA survey comprised 9 parts: respondents' basic information, IT application, infrastructure and hardware use, information system application, IT outsourcing, IT construction obstacles, information system construction investment, data standardization, and regional medical and health information system construction. We mainly used the data from the first to seventh of the 9 parts; in particular, the data from parts I-V and VII: respondents' basic information, IT application, infrastructure and hardware use, information system application, IT outsourcing, and information system construction investment. Each year’s survey report provides a summary of the current situation of hospital digitalization and the overall trend of HIT in China. The 2018 survey was completed between March 2019 and June 2019 and released on September 10, 2019.

### Research Subjects: Hospital Information Technology Department–Related Attributes of the China Hospital Information Management Association Annual Survey

In China, most hospitals purchase HIT software from the HIT market, which is outsourced by system suppliers. Therefore, the IT departments of hospitals are mainly responsible for the procurement, management, and subsequent maintenance of the system. The head of the IT department is the CIO of the hospital, and these CIOs are the main subjects of this research.

### Technology Diffusion Model and Bass Modeling

Bass diffusion modeling was employed as one method to predict the progress of EMR adoption and analyze its characteristics. Diffusion theory is an essential branch of communication theory that has long attracted the attention of scholars in management, marketing, and other disciplines [11]. The Bass model has been widely used in the application and forecasting analysis of new products and technologies [12,13], including many medical-related technologies [14-16]. The Bass model has 9 key assumptions [13,16], most of which satisfy the scenarios of this study (eg, market potential of the new product remains constant over time, the geographic boundaries of the social system do not change over the diffusion process).

**Figure 1:**
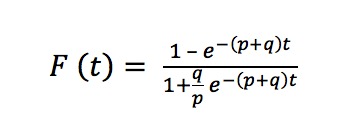
Mathematical expression of the Bass model.

There are two important measures for the implementation of the Bass model [17]. The external influence coefficient is called the *innovation* effect, represented as the p-coefficient. It corresponds to the probability of using the products under the influence of public media or other external factors among users who have not used the product. The internal influence coefficient refers to the *imitation* effect and is expressed as the q-coefficient. This effect depicts the probability of the same users who would begin to use the product under the influence of peers who have already used the product [18]. The mathematical expression of the Bass model is shown in Figure 1, where M is the potential market, F(t) is the portion of M that have adopted by time t, p is the coefficient of innovation, and q is the coefficient of imitation.

We conducted statistical analyses and forecasts using linear optimization in Excel for Mac 2011 (Microsoft Corp). The parameters of the Bass model were trained and estimated using SPSS Statistics software version 20 (IBM Corp). We used the method of least squares to determine the optimal values of q and p.

## Results

### Descriptive Analysis

#### Scale and Coverage of Research

**Figure 2:**
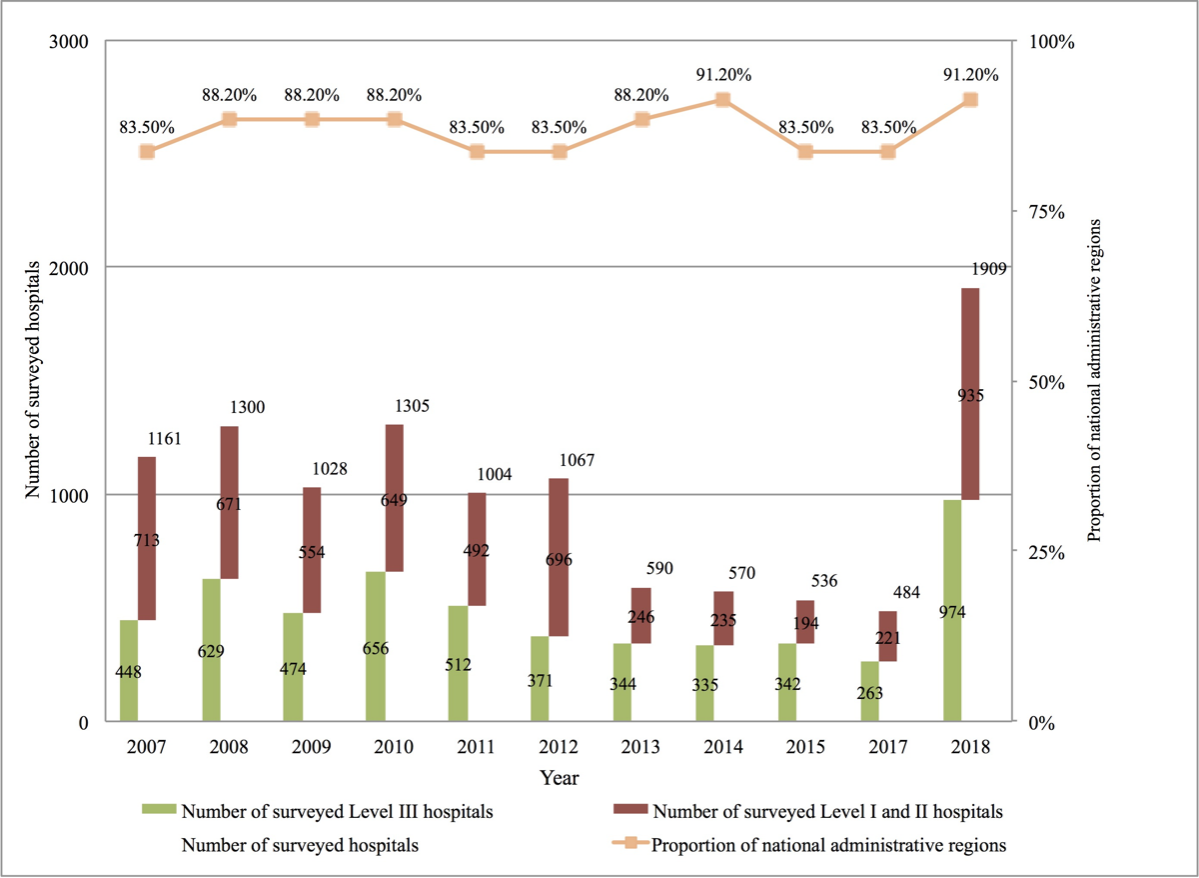
China Hospital Information Management Association survey on hospital digitalization in China by hospital level, 2007 to 2018.

The scale and regional coverage of the 2007-2018 CHIMA annual survey of hospital information systems are shown in Figure 2 below. In China, all hospitals are categorized by a government board into three levels: primary (roughly equivalent to community-based health centers in the United States), secondary (county- and municipal-level health care facilities), and level III (large, advanced general or specialty hospitals, often academic medical centers) [19]. In this study, hospitals were divided into two categories: level III and ≤ secondary.

#### Hospital Health Information Technologies Human Resources: Quantity, Quality, and Work Stress

**Figure 3:**
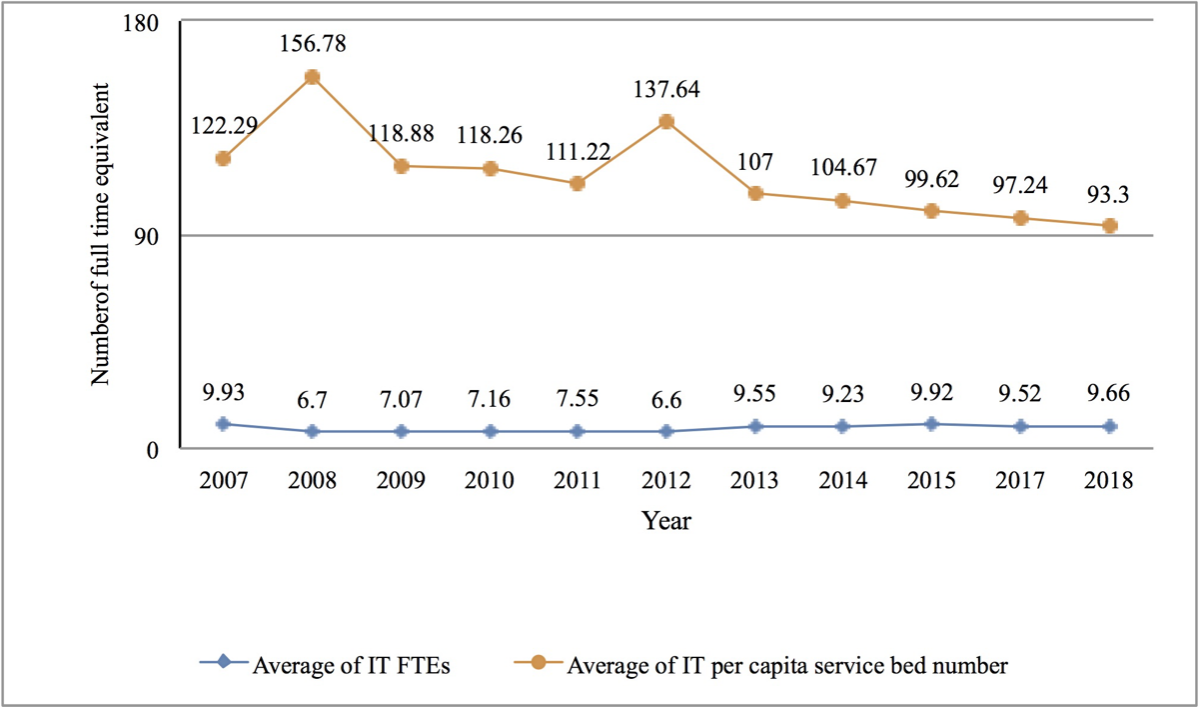
Proportion of human resources in China’s hospital information technology centers from 2007 to 2018. FTE: full-time equivalent; IT: information technology.

**Figure 4:**
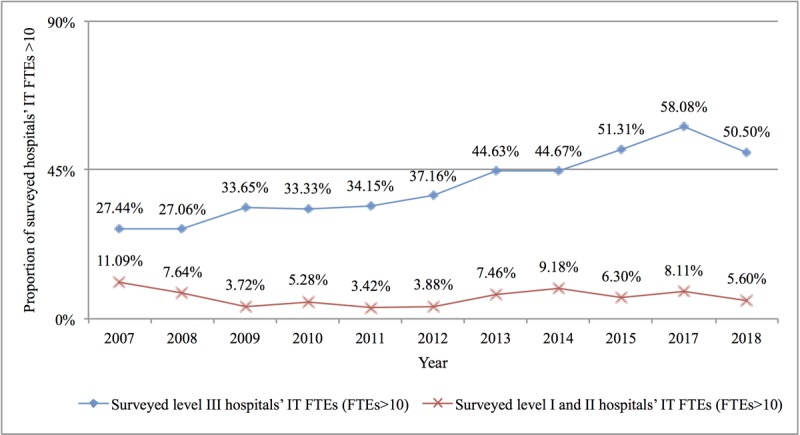
Information technology centers with 10 or more staff members in China’s level III hospitals and hospitals below level III from 2007 to 2018. IT: information technology; FTE: full-time equivalent.

**Figure 5:**
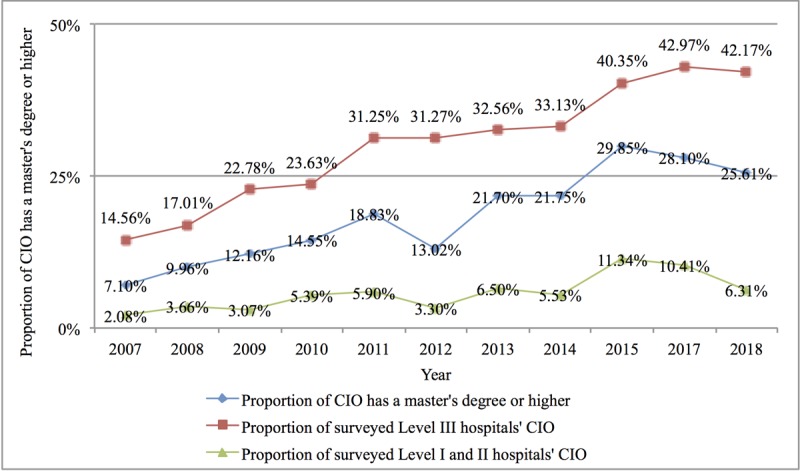
Proportion of chief information officers with a master’s degree or above in China’s hospital information technology centers from 2007 to 2018. CIO: chief information officer.

**Figure 6:**
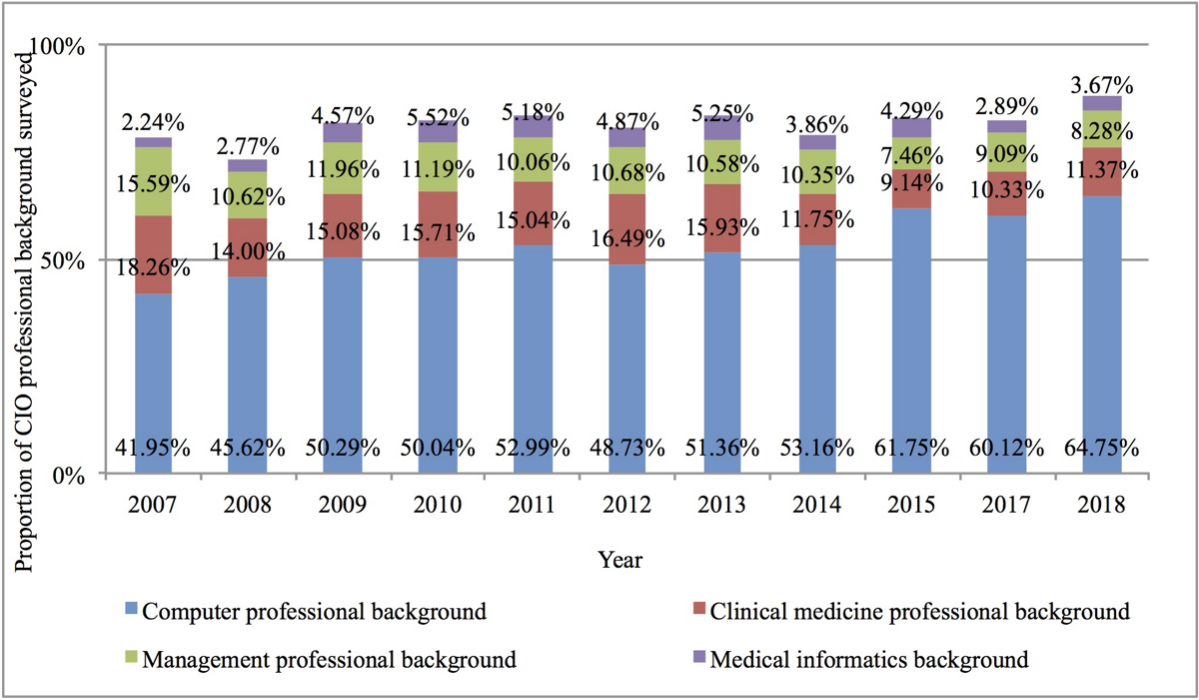
Academic background and composition of chief information officers in China’s hospital information technology centers from 2007 to 2018. CIO: chief information officer.

From 2007 to 2018, the shortage of human resources in China’s hospital IT centers eased and the quality of personnel improved (Figures 3-6).

First, manpower allocation was 9.66 full-time equivalents (FTEs), on average, in 2018. At the same time, the average number of beds managed by each staff member in the hospital IT center decreased from 122 in 2007 to 93 in 2018, as shown in Figure 3. The proportion of IT centers in level III hospitals with 10 or more staff members increased from 27.44% in 2007 to 50.50% in 2018, as shown in Figure 4. However, compared with their US counterparts, the gap was still significant. According to the HIMSS annual survey data, as early as 2006, more than 80% of IT centers in US hospitals were staffed with more than 10 people [20].

Second, the professional quality of CIOs in China’s hospital IT centers also improved significantly. The proportion of hospital CIOs with a master’s degree or above nearly tripled from 7.1% in 2007 to 25.61% in 2018. The proportion of CIOs with a master’s degree in level III hospitals increased from 14.56% in 2007 to 42.17%, and the proportion of CIOs with a master’s degree in level I and II hospitals increased from 2.08% to 6.31%, as shown in Figure 5. The proportion of CIOs with medical-related backgrounds in China’s hospital IT centers was very low and even showed a downward trend, falling from 18.25% in 2007 to 11.37% in 2018, while computer majors became mainstream, rising from 41.95% in 2007 to 64.75% in 2018. As the counterpart discipline of HIT, medical informatics is in a marginally weak position among the background disciplines of CIOs in hospital IT centers, rising only from 2.24% in 2007 to 3.67% in 2018 (see Figure 6 for details).

#### Hospital Health Information Technologies Investment

Stimulated and driven by the state’s direct investment and relevant policies, the total direct investment by hospitals in HIT greatly increased.

**Figure 7:**
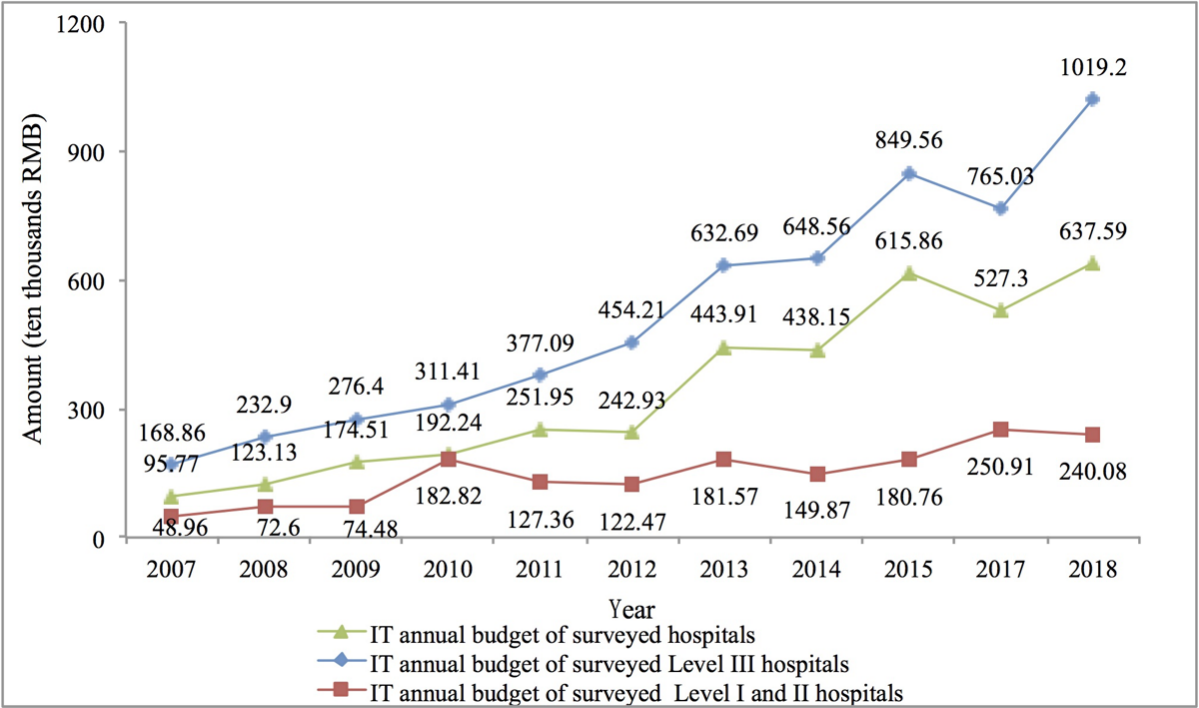
Health information technologies investment in Chinese hospitals from 2007 to 2018. IT: information technology.

First, the total investment in HIT rose from ¥957,700 (US $136,875) per year in 2007 to ¥6.376 million (US $0.91 million) per year in 2018, an increase of 5.66 times. The average annual HIT investment of level III hospitals increased from ¥1.689 million (US $0.24 million) per year to ¥10.192 million (US $1.46 million) per year, an increase of 5 times. The average annual HIT investment of hospitals below level III increased from ¥489,600 (US $69,974) to ¥2.401 million (US $0.34 million) per year, an increase of nearly 4 times, as shown in Figure 7.

**Figure 8:**
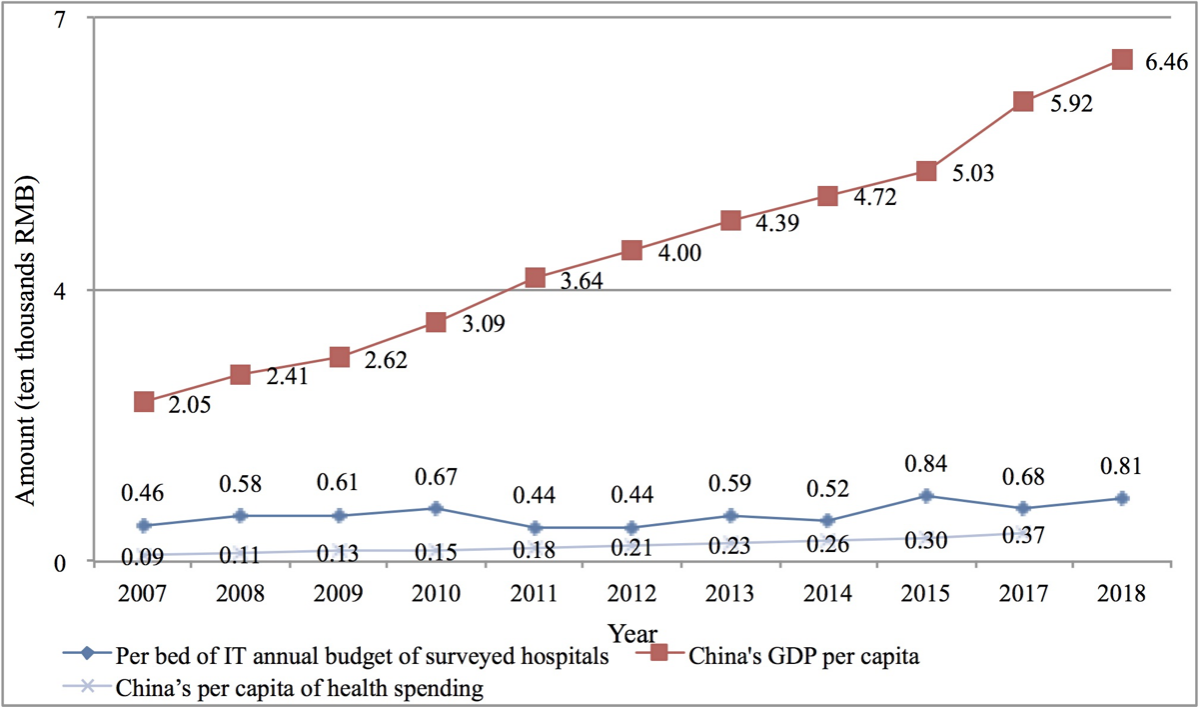
China’s per capita gross domestic product, medical expenditure per capita, and information technology investment per hospital bed from 2007 to 2018. IT: information technology; GDP: gross domestic product. (Note: As of the date of submission, the per capita health spending data for China in 2018 has not been announced.).

Second, relative to China’s fast-growing economy (per capita gross domestic product increased from ¥20,500 (US $2930) in 2007 to ¥64,600 (US $9233) in 2018, an increase of 2.15 times) and the rapid increase of medical expenses (per capita medical expenses increased from ¥900 (US $129) in 2007 to ¥3700 (US $528) in 2017, an increase of 4.28 times), the annual IT investment per bed increased insignificantly (only 76%) from ¥4600 (US $657) in 2007 to ¥8100 (US $1158) in 2018, as shown in Figure 8. However, due to the marginal cost of software and service products, the higher the base number of users, the larger the market, and the lower the cost of digitalization allocated to each single service object (bed). We believe that even considering the inflation factor, the connotation of the digitalization investment of ¥8100 (US $1158) per bed in 2018 was much greater than that of ¥4600 (US $657) in 2007.

#### Hospital Network Environment Support

**Figure 9:**
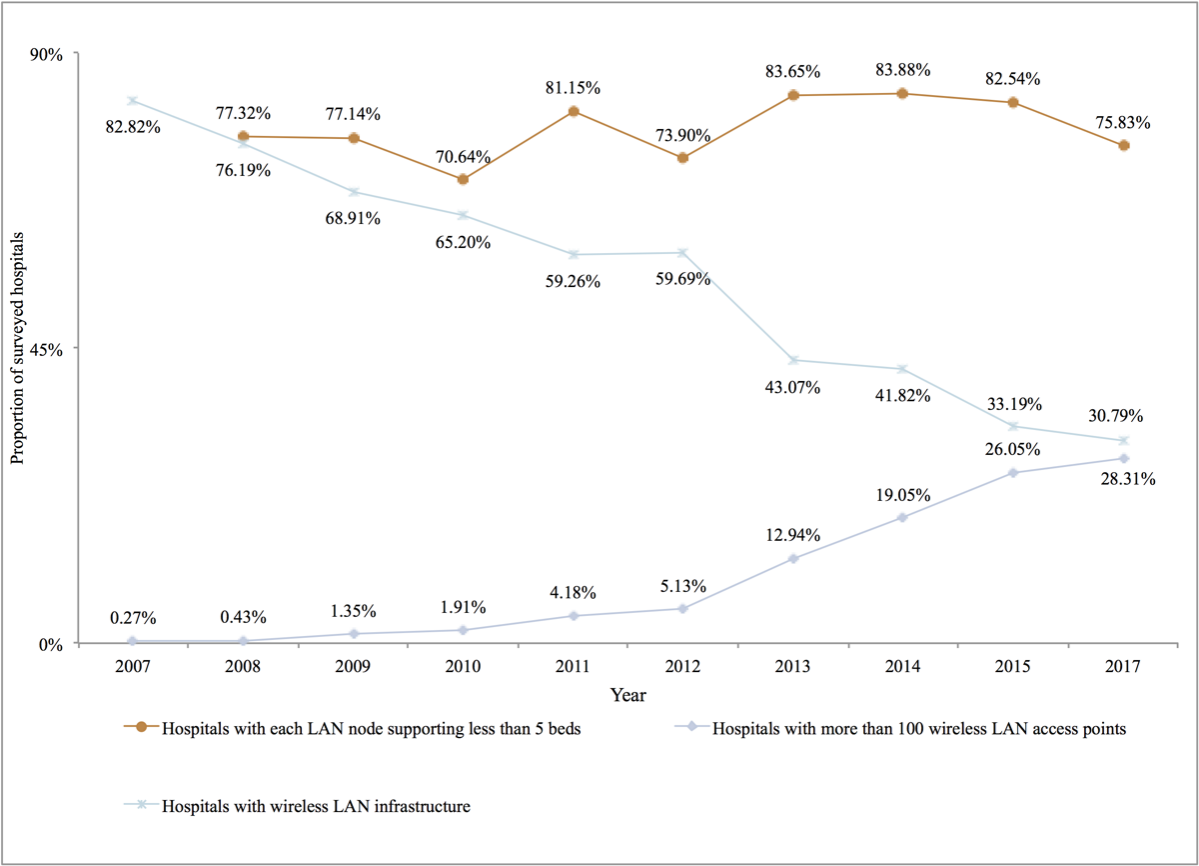
Wired local area network and wireless network facility construction in Chinese hospitals from 2007 to 2017. LAN: local area network. (Note: Wireless network-related indicators were not included in the 2007 CHIMA Annual Survey; relevant indicators on hospital networks were no longer included in the 2018 CHIMA Annual Survey.).

The overall network infrastructure construction and configuration of Chinese hospitals have also been continuously improving. On one hand, in terms of traditional wired Ethernet local area network (LAN) construction, in 2017 about 75.83% of the sampled hospitals had achieved the goal of one wired LAN interface supporting 5 beds or fewer, which was basically the same as in 2008. On the other hand, in terms of wireless network infrastructure, about 69.21% of the sampled hospitals had launched wireless networks, compared with 17.18% in 2007. In addition, about 30.79% of the sampled hospitals that had launched wireless networks had more than 100 wireless network access hotspots in 2017, as shown in Figure 9.

#### Implementation and Application of Clinical Information Systems (Including Electronic Medical Records) in Chinese Hospitals

CIS has been implemented in Chinese hospitals to a considerable extent. After more than 10 years of development, medical digitalization has been adopted as one of the “four beams and eight pillars” supporting China’s health care reform, especially China’s second health care reform, which began in 2010; a large amount of funds and resources have been invested, and a large number of policies have been promulgated for support and guidance [21]. Under this stimulus, from 2007 to 2018, the utilization rate of major CIS systems (including computerized prescriber order entry [CPOE], laboratory information systems [LIS], picture archiving and communication systems [PACS], and EMR) in sampled hospitals increased significantly.

**Figure 10:**
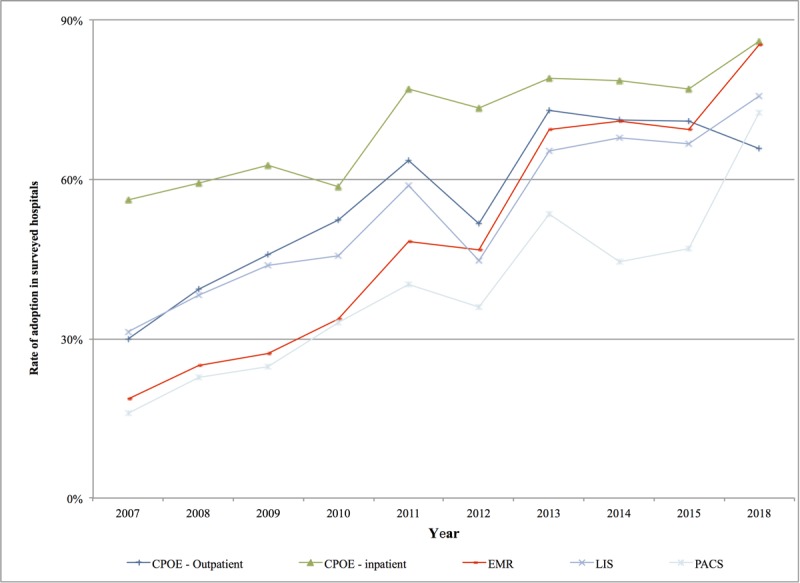
Application and implementation of computerized prescriber order entry, electronic medical record, laboratory information system, and picture archiving and communication system in the sampled hospitals from 2007 to 2018. CPOE: computerized prescriber order entry; EMR: electronic medical record; LIS: laboratory information system; PACS: picture archiving and communication system. (Note: Due to a change in the leadership of CHIMA in 2016, the CHIMA Annual Survey was not launched, and survey data of 2016 and 2017 were not available for analysis.).

CIS has been applied to a considerable extent, and the popularization rate of EMR exceeded the average level of its US counterparts in 2015 [22] (85.26% vs 83.8%) and the average level of its German counterparts in 2017 [23] (85.26% vs 68.4%). CPOE outpatient services rose from 30% in 2007 to 65.9% in 2018, CPOE inpatient services rose from 56.1% in 2007 to 85.9% in 2018, EMR rose from 18.6% in 2007 to 85.3% in 2018, LIS rose from 31.3% in 2007 to 75.7% in 2018, and PACS rose from 15.9% in 2007 to 72.5% in 2018, as shown in Figure 10. The construction and implementation of CIS including CPOE, EMR, LIS, and PACS in level III hospitals in China has developed vigorously and is maturing daily. In the 2018 survey, the utilization rates of CPOE, EMR, LIS, and PACS in the sampled hospitals all exceeded 65%. China’s hospital digitalization focuses on the construction of a patient-centered clinical information system that directly serves medical personnel and provides strong support for health care reform.

### Bass Model Forecast Analysis: Development Trends of Electronic Medical Records in Chinese Hospitals

We estimated the p- and q-coefficients using the Bass model and linear optimization based on the CHIMA hospital adoption rate of EMR data from 2007-2018 (excluding 2016 and 2017; because of a leadership change in CHIMA in 2016, the 2016 annual survey was not launched). Table 1 describes the parameter estimation results in the final model, which indicates that the Bass model fit the CHIMA dataset well.

**Figure 11:**
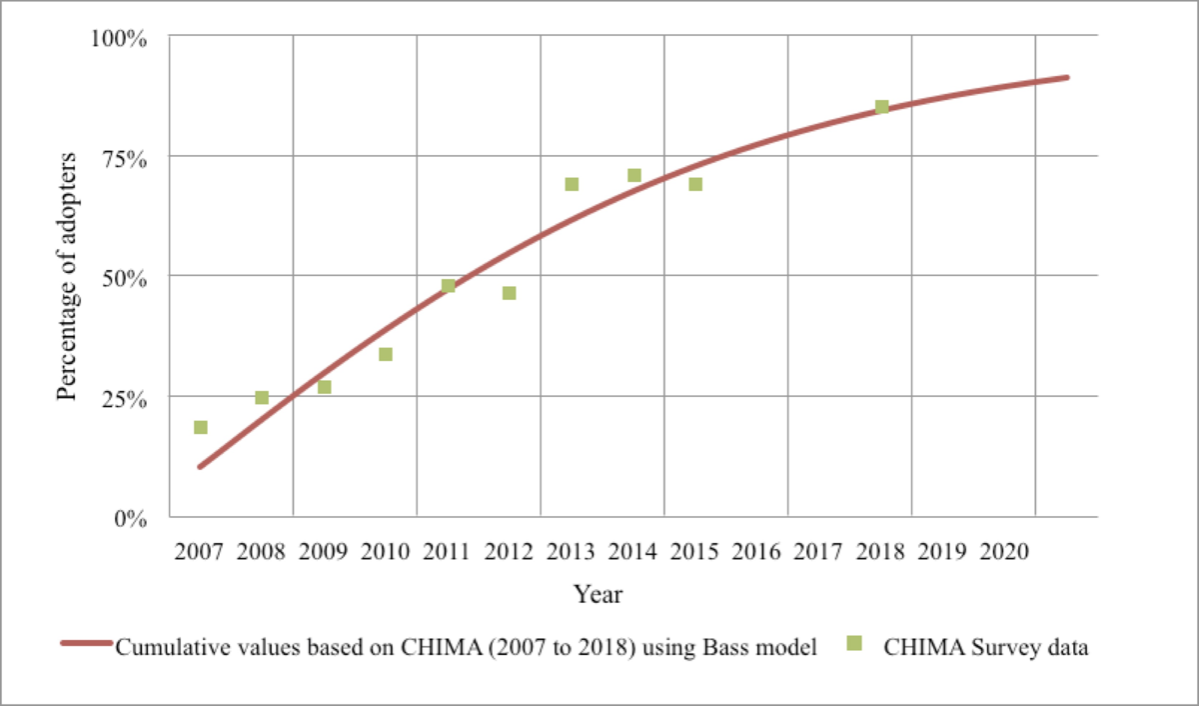
Electronic medical record adoption among Chinese hospitals using the China Hospital Information Management Association Annual Survey (figures for 2016, 2017, and 2020 are forecasted using the Bass mode). CHIMA: China Hospital Information Management Association.

Figure 11 shows the fit of the EMR popularization data of Chinese hospitals from 2007-2018 (excluding 2016 and 2017), and assuming that there will be no major policy adjustments and technological upgrades in the future, the forecast of EMR popularization in hospitals by 2020 has the adoption rate of EMR expected to reach about 91.4%. Although great progress has been made, there is still a slight gap compared with US counterparts. According to research by Jha et al [24], the adoption of certified EHR systems among US nonfederal acute care hospitals in 2020 is expected to be close to 100%.

**Table 1:** The estimating parameters for Chinese hospitals’ adoption rate of electronic medical records.

Model parameter	Estimated result
External motivation coefficient (p)	0.102
Internal motivation coefficient (q)	0.106
Motivation coefficient ratio (q/p)	1.039
*R^2^*	.951

On one hand, the fitted external coefficient (p=0.102) of Chinese hospitals’ adoption rate of EMR is much larger than those of medical examination equipment popular in the United States, such as ultrasound images (p=0.000) and molybdenum target x-rays (p=0.000) [25]. On the other hand, it is relatively small compared with those of other consumer electronic products that provide information support, such as electronic calculators (p=0.143) and personal computers (p=0.121) [26]. At the same time, we found that for China’s current medical system to join the regional medical care alliance, hospitals would need to invest significant manpower and material resources to build IT infrastructure and transform traditional paper-based medical processes and care, as well as the communication methods and business processes between doctors and patients, so as to provide medical services more effectively and efficiently. From this perspective, EMR may slow the spread of the universal technologies, such as personal computers and electronic calculators, that have been widely publicized. Despite this, the implementation and promotion of EMR have still been effectively advanced under the strong support and publicity of China’s administrative supervision and public media.

On the other hand, compared with certain medical examination equipment, the internal motion coefficient (q) fitted in this study is relatively small, which indicates that the internal driving force of the hospitals themselves was relatively weak in this process. First, according to research by Sillup et al [25], the Bass model fitting parameter of ultrasound images in the United States was q=0.510 and the model fitting parameter of molybdenum target x-ray data was q=0.738, while in our study, q is only 0.106. Second, we believe that it cannot be immediately clear how much benefit hospitals can directly create for doctors and patients from the actual use of EMR. In many cases, the government took the lead, the public media and public opinions promoted it, and HIT technology, including EMR and CIS, was used to realize the artificial project of sharing medical resources in the hospital with information as the link rather than being a spontaneous product of the hospital.

## Discussion

### Summary

This study uses the data from the survey of medical digitalization construction conducted by CHIMA, a national industrial association in China, on 10,954 Chinese hospital CIOs from 2007 to 2018 to evaluate the progress of HIT in Chinese hospitals in terms of professional staffing, funding, infrastructure construction, and clinical system application. Here we discuss the US HIMSS annual survey exploring the difficulties and challenges encountered in the development of China’s HIT.

### Constraints on Health Information Technologies Human Resources

As of 2018, compared with their US counterparts, IT departments in Chinese hospitals were still short of IT human resources. The average allocation of human resources in the IT centers of the sampled Chinese hospitals was only 28% of that of their US counterparts in 2014 (9.66 FTEs vs 34 FTEs). We believe that this may further affect the development and deepening of subsequent HIT applications. In terms of the quantity of human resources, the survey results showed that hospital IT centers had an average of 9.66 FTEs in 2018, and the number of beds served by each IT staff member also dropped from 122 in 2007 to 93 in 2018. However, according to the annual survey of HIMSS in 2014, IT centers in the United States were equipped with an average of 34 FTEs, 3.5 times that of their Chinese counterparts [27]. As early as 2006, more than 80% of the IT centers in US hospitals were equipped with 10 or more FTEs [20], while in China, by 2018, level III hospitals with 10 or more FTEs accounted for 50.5%, and hospitals classified as level II and below with 10 or more FTEs accounted for only 5.6%. Based on the results of the Information Statistics Center of the National Health Commission of the People’s Republic of China in 2006, 10 to 30 HIT professionals were required for each level III hospital (600 beds or more), 6 to 15 for each level II hospital (300 to 600 beds), and 3 to 6 for each level I hospital (100 to 300 beds) [28].

Hospital information work such as system management; operation and maintenance; system and network security; content management; system integration and interface design; and hardware, network, and software maintenance is tedious and labor intensive, especially providing training for users of various levels and types of systems. Considering that many of the above services need to be provided on a 24/7 basis, it is an objective need and an inevitable trend for the development of hospital information systems to consume a large amount of human resources. We believe that, on one hand, the breadth and depth of HIT application in Chinese hospitals are still relatively low; on the other hand, policy makers and hospital managers do not fully understand that the safe and effective operation of information systems depends on the support of a large number of human resources.

The analysis of the highest degree of CIOs in hospitals indicates that the educational levels of information professionals working in hospitals in China had significantly improved; however, their distribution was not uniform. In 2018, 25.6% of CIOs had a master’s degree or above, an increase of 18.51% compared with 2007; however, there was a significant difference between level III hospitals and hospitals below level III. Taking 2018 as an example, the proportion in the former was 35.8% higher than that in the latter. We believe that IT faces the urgent matter of cultivating interdisciplinary senior management talent who understand both medical care and IT technology. According to the survey results in 2018, more than 60% of the CIOs in China’s hospital IT centers majored in computer information systems, while only 3.67% had a medical informatics background. Hospital CIOs demonstrated a relative lack of knowledge of hospital information management and medical informatics.

Unlike the cross-disciplinary definition of “using computer technology in the fields of health care and medical science” [29] in the United States, the medical informatics discipline in China is very young; however, it is gradually rising with the development of hospital digitalization in China on the basis of library science [30]. It was not formally established as an independent discipline until 2010, and at present, very few educational institutions in China have medical information research institutes or postgraduate programs (27 master’s degree programs and 5 doctoral degree programs), and most of the current students are undergraduates who cannot meet the business needs of hospitals [31]. We suggest that reeducating experts interested in hospital digitalization in current leading positions in Chinese hospitals at all levels (systematically supplementing their knowledge of medical informatics based on International Medical Interpreters Association’s training syllabus) and granting certificates to qualified personnel may be a shortcut to cultivating the required talent [32].

### Hospital Health Information Technologies Investment Trends

HIT investment in a large number of hospitals classified as level II and below in China may be mainly driven by state investment, but their own investment willingness is not strong. After the previous health care reform, hospitals could only receive limited government financial subsidies and had to be self-financing [33]. Therefore, their financial strength was very limited. Beginning in 2010 (Figure 7), the average investment in HIT in Chinese hospitals increased rapidly, from ¥1.9224 million (US $0.27 million) in 2010 to ¥6.3759 million (US $0.91 million) in 2018, an increase of nearly 2.32 times. However, we found that the increase was extremely uneven (ie, after 2010, the HIT investment growth rate of level III hospitals was much higher than that of level II and below hospitals, and this imbalance may have caused new imbalances in medical resources).

According to an analysis of national HIT investment directions from 2010 to 2015 (Multimedia Appendix 1), the investment targets were mainly level II and below hospitals. According to the survey results, HIT investments in such hospitals in 2007, 2008, and 2009 were only ¥489,600 (US $69,974), ¥726,000 (US $103,760), and ¥744,800 (US $106,447), respectively. After 2010, HIT investment increased to ¥1.82 million (US $0.26 million), but subsequent growth was weak, with an increase of only ¥572,600 (US $81,836). During the same period, the increase for level III hospitals was ¥7.0779 million (US $1.01 million), which was 11.3 times the former. We suggest that the issue of how to raise the awareness of the majority of primary-level hospital leaders of the dividend that HIT brings to hospital development is one of the areas for which the National Health Commission should formulate relevant HIT development policies in the next stage.

### Rapid Development of Electronic Medical Records in China and Difficulties in Recycled Use of Precipitated Data

The utilization rate of CIS represented by EMR in Chinese hospitals continued to increase. First, the EMR popularization rate of the sampled hospitals increased from 18.6% in 2007 to 85.3% in 2018, an increase of 3.6 times in 8 years, and the average EMR implementation rate of the sampled hospitals exceeded the average level of their US counterparts in 2015 [22] and their German counterparts in 2017 [23] (85.26% vs 83.8% vs 68.4%, respectively). Considering that as of 2017, the number of various medical institutions in China was more than 27,700, while that in the United States was more than 6300, the former close to 4.5 times of the latter, the growth rate was already considerable. Second, based on the Bass model fitting results of EMR utilization rate data from the sampled hospitals in the 2007-2015 CHIMA annual surveys, it is suggested that this growth was largely driven by external motivation coefficient effects (p-coefficient). That is, hospitals began to use EMR to a large extent under the influence of external administrative forces. The specific manifestation was p≈q (p=0.102, q=0.106), which is consistent with the Chinese government’s attitude and strategy toward HIT development. We believe that the development mode of China’s medical industry, which accepts government instruction, uses unified planning of administrative intervention, and enables HIT to achieve leapfrog improvements in a short period of time, is one of the most important and unique contributions of China’s HIT.

On the other hand, as CIS, represented by EMR, has gradually been built and put into use, it faces the challenge of how to carry out the secondary application of massive precipitated data in China. In the survey samples in 2018, the implementation rates of CPOE, EMR, LIS, and PACS all exceeded 65%. However, real-world clinical data from EMR and other CIS have not been widely used for secondary data research in China. The second health care reform in China established medical digitalization as an essential strategic development direction [34,35]. The reform also set the long-term goal of building and improving HIT, especially EMR software infrastructure in various hospitals. Based on the research feedback, the coverage of EMRs within hospitals reached 80% in 2018, which exceeds the coverage rate of hospitals in the United States in 2015. However, the interoperability, quality, and ease of use of EMR data are lacking.

In terms of interoperability, the various EMR systems used in different hospitals are incompatible with each other. There are currently more than 300 EMR software providers in China, all with their own proprietary technology structures and data standards. The hospitals have no initiative to exchange data despite the government establishment of some regional health information organizations (RHIOs). As of 2015, the proportion of hospitals participating in RHIOs in the sample had reached 50% [36]. Nevertheless, most of them are in the initial stages and are far from interoperability due to semantic problems.

Concerning the quality of information, EMR data in China is not informative. One study used Charmaz’s grounded theory approach to perform a difference analysis of the medical questions and number of examination and treatment terminologies in the EMR corpus samples among 3 US hospitals and a Chinese hospital [37]. The study found that in certain types of medical records, the density of technical terms in Chinese EMRs was much lower than that in English EMRs. Chinese EMRs contained only half the amount of technical terms compared to US EMRs, indicating that the latter is more professional. We believe that this may be due to the more complicated and rigorous legal environment in the United States, where more complete and comprehensive examinations and discussions with patients are required to prevent medical disputes.

Regarding ease of use, there are large discrepancies and gaps between EMR data in China and the United States. This indirectly leads to problems of integrity and accuracy in China’s EMR data. Previous research used the US Stage 2 Meaningful Use objectives to evaluate usability of EMR data from the two best Chinese teaching hospitals affiliated with Peking University Medical School (Peking University First Hospital and Beijing Cancer Hospital) [38]. They found that only 50% of the Meaningful Use targets were supported in the EMRs of Chinese hospitals. Moreover, the Chinese hospitals still used many paper forms to augment the clinical work despite the establishment of EMRs, resulting in a considerable loss of clinical information beyond the EMR system. The ease of use of EMRs at Peking University First Hospital and Beijing Cancer Hospital [39] was examined based on the standard of the Unified Framework For EHR Usability [40], and a total of 85 problems in usability relevant to clinical tasks were found, some of which may even seriously affect the quality and safety of medical services.

### Limitations

This study is based on self-reported questionnaire survey results from 2007-2018 regarding investment in HIT funds, staffing and training, investment in funds, construction and implementation of applied technologies, and difficulties encountered in the processes of Chinese hospitals. The data have not been independently verified. Therefore, such an analysis is subject to the potential confounding factors of data bias. In addition, we did not use a multivariate model to evaluate the independence of different factors (such as hospital level, hospital type, and economic development level in the region of the hospital). Although we only limit the inference to our own samples, these analyses are still valuable because these data spanning 12 years are the only data on the development trend of HIT in China collected by China’s national industrial association that can be quantitatively analyzed.

In addition, the absence of feedback on data offset will affect the survey results. For example, hospitals with high HIT application levels are more likely to give feedback. However, the feedback providers of this survey should be representative of the true level of HIT application in Chinese hospitals to some extent, especially for the level III hospitals in China, which have an average coverage rate of 34.44% over 12 years.

### Conclusions

China’s unique institutional model may have distinct advantages in achieving the goals of health care reform. In this case, the Chinese government used a top-down, top-level design mode and took HIT development as an important technical support and starting point to support health care reform through policies, systems, funds, and other comprehensive methods. According to the survey results of the CHIMA annual survey of hospital information systems, with about only one-fifth of the required funding and one-fourth of the required human resources funding per hospital IT FTE as compared with the US HITECH project, China’s EMR coverage in 2018 exceeded the average level of its US counterpart in 2015 and the average level of its German counterpart in 2017. Fitting results based on the Bass model suggest that it is expected that 91% of hospitals in China will use EMR by 2020. All signs show that the Chinese government is gradually approaching and realizing the phased goals set in the second health care reform launched in 2010: integrating medical resources, improving medical care popularization, reducing medical costs, and improving medical care quality.

## Appendix

Multimedia Appendix 1 National health information technologies investment directions from 2010 to 2015.

## Abbreviations

CHIMA: China Hospital Information Management Association
CIS: clinical information system
CIO: chief information officer
CPOE: computerized prescriber order entry
EHR: electronic health record
EMR: electronic medical record
FTE: full-time equivalent
HIMSS: Healthcare Information and Management Systems Society
HIT: health information technologies
HITECH: Health Information Technology for Economic and Clinical Health
IT: information technology
LAN: local area network
LIS: laboratory information system
PACS: picture archiving and communication system
RHIO: regional health information organization

## References

[1] Kim Y, Jung K, Park Y, Shin D, Cho S, Yoon D, Park RW (2017). Rate of electronic health record adoption in South Korea: a nation-wide survey. Int J Med Inform.

[2] Mennemeyer ST, Menachemi N, Rahurkar S, Ford EW (2016). Impact of the HITECH Act on physicians' adoption of electronic health records. J Am Med Inform Assoc.

[3] Gold M, McLaughlin C (2016). Assessing HITECH implementation and lessons: 5 years Later. Milbank Q.

[4] Sheikh A, Jha A, Cresswell K, Greaves F, Bates DW (2014). Adoption of electronic health records in UK hospitals: lessons from the USA. Lancet.

[5] Lei J, Wen D, Zhang X, Li J, Lan H, Meng Q, Sittig DF (2017). Enabling health reform through regional health information exchange: a model study from China. J Healthc Eng.

[6] Zhu C (2015). Healthy China 2020 Strategic Research Report. 1st Edition.

[7] (2018). State Council, the People's Republic of China.

[8] Kruse CS, Beane A (2018). Health information technology continues to show positive effect on medical outcomes: systematic review. J Med Internet Res.

[9] Adler-Milstein J, DesRoches CM, Furukawa MF, Worzala C, Charles D, Kralovec P, Stalley S, Jha AK (2014). More than half of US hospitals have at least a basic EHR, but stage 2 criteria remain challenging for most. Health Aff (Millwood).

[10] China Hospital Information Management Association.

[11] Bass FM (2004). A new product growth for model consumer durables. Manag Sci.

[12] Van den Bulte C (2002). Want to know how diffusion speed varies across countries and products? Try using a Bass model. PDMA visions.

[13] Sood A, James GM, Tellis GJ, Zhu J (2012). Predicting the path of technological innovation: SAW vs. Moore, Bass, Gompertz, and Kryder. Market Sci.

[14] Kharrazi H, Gonzalez CP, Lowe KB, Huerta TR, Ford EW (2018). Forecasting the maturation of electronic health record functions among US hospitals: retrospective analysis and predictive model. J Med Internet Res.

[15] Ford EW, Hesse BW, Huerta TR (2016). Personal health record use in the United States: forecasting future adoption levels. J Med Internet Res.

[16] Norton JA, Bass FM (1987). A diffusion theory model of adoption and substitution for successive generations of high-technology products. Manag Sci.

[17] Mahajan V, Muller E, Bass FM (2018). New product diffusion models in marketing: a review and directions for research. J Marketing.

[18] Grimm SE, Stevens JW, Dixon S (2018). Estimating future health technology diffusion using expert beliefs calibrated to an established diffusion model. Value Health.

[19] Lei J, Guan P, Gao K, Lu X, Chen Y, Li Y, Meng Q, Zhang J, Sittig DF, Zheng K (2014). Characteristics of health IT outage and suggested risk management strategies: an analysis of historical incident reports in China. Int J Med Inform.

[20] PricewaterhouseCoopers LLP (2007). California HealthCare Foundation.

[21] Lei J, Meng Q, Li Y, Liang M, Zheng K (2016). The evolution of medical informatics in China: a retrospective study and lessons learned. Int J Med Inform.

[22] Charles D, Gabriel M, Searcy T (2015). Adoption of electronic health record systems among US non-federal acute care hospitals: 2008-2014.

[23] Esdar M, Hüsers J, Weiß J, Rauch J, Hübner U (2019). Diffusion dynamics of electronic health records: a longitudinal observational study comparing data from hospitals in Germany and the United States. Int J Med Informatics.

[24] Adler-Milstein J, Holmgren AJ, Kralovec P, Worzala C, Searcy T, Patel V (2017). Electronic health record adoption in US hospitals: the emergence of a digital. J Am Med Inform Assoc.

[25] Sillup GP (1992). Forecasting the adoption of new medical technology using the Bass model. J Health Care Mark.

[26] Ford EW, Menachemi N, Phillips MT (2006). Predicting the adoption of electronic health records by physicians: when will health care be paperless?. J Am Med Inform Assoc.

[27] (2014). Healthcare Information and Management Systems Society.

[28] Luo S, Zhang K, Li B (2010). Medical informatics in China: healthcare IT trends, academic and research developments. Yearb Med Inform.

[29] Musen M, Bemmel H (2002). Handbook of Medical Informaties.

[30] Liang J, Wei K, Meng Q, Chen Z, Zhang J, Lei J (2017). The gap in medical informatics and continuing education between the United States and China: a comparison of conferences in 2016. J Med Internet Res.

[31] Liang J, Wei K, Meng Q, Chen Z, Zhang J, Lei J (2017). Development of medical informatics in China over the past 30 years from a conference perspective and a Sino-American comparison. PeerJ.

[32] Lau F (2007). Distributed health informatics graduate education for working professionals. Int J Med Inform.

[33] Editorial (2010). Chinese doctors are under threat. Lancet.

[34] Deng H, Wang J, Liu X, Liu B, Lei J (2018). Evaluating the outcomes of medical informatics development as a discipline in China: a publication perspective. Comput Methods Programs Biomed.

[35] Jia Y, Wang W, Liang J, Liu L, Chen Z, Zhang J, Chen T, Lei J (2018). Trends and characteristics of global medical informatics conferences from 2007 to 2017: a bibliometric comparison of conference publications from Chinese, American, European and the Global Conferences. Comput Methods Programs Biomed.

[36] Liang J, Zheng X, Chen Z, Dai S, Xu J, Ye H, Zhang Z, Ge F, Lei J (2019). The experience and challenges of healthcare-reform-driven medical consortia and Regional Health Information Technologies in China: a longitudinal study. Int J Med Inform.

[37] Wu Y, Lei J, Wei W, Tang B, Denny JC, Rosenbloom ST, Miller RA, Giuse DA, Zheng K, Xu H (2013). Analyzing differences between chinese and english clinical text: a cross-institution comparison of discharge summaries in two languages. Stud Health Technol Inform.

[38] Lei J, Sockolow P, Guan P, Meng Q, Zhang J (2013). A comparison of electronic health records at two major Peking University Hospitals in China to United States meaningful use objectives. BMC Med Inform Decis Mak.

[39] Xu L, Wen D, Zhang X, Lei J (2016). Assessing and comparing the usability of Chinese EHRs used in two Peking University hospitals to EHRs used in the US: a method of RUA. Int J Med Inform.

[40] Zhang J, Walji MF (2011). TURF: toward a unified framework of EHR usability. J Biomed Inform.

