# Supplementary material for: Evaluating the Applications of Health Information Technologies in China During the Past 11 Years: Consecutive Survey Data Analysis
Source: JMIR Med Inform. 2020 Feb 10;8(2):e17006. doi: 10.2196/17006 (PMC7055786; doi:10.2196/17006)
Supplement: Multimedia Appendix 1 [file medinform_v8i2e17006_app1.docx]

Appendix

Table 1 Summary of major HIT investments from 2010 to 2015 in China

| Investment orientation | Investment quantity | Achievements made |
| --- | --- | --- |
| To support grass- rooted medical and health institution management information system construction, and rural health room informatization in Middle and West China. | 1.5 billion dollars | Health information system networks covering administrative villages were preliminarily built. EHR medical informatization was promoted in all 434505 administrative villages in 24 provinces/ cities in Middle and West China; hospital informatization upgrading was promoted in 2264 county-level first-class hospitals in 24 provinces/cities in Middle and West China. Telemedicine centers were built in 5 representative counties (totally 110 counties) in each of 24 provinces/cities in Middle and West China; EHR regional information platforms were set up in Anhui, Chongqing and Xinjiang. Overall medical informatization upgrading was promoted in 16 pilot cities selected for public hospital pilot reform; Medical informatization was upgraded in high-end 3A hospitals in the 24 provinces/cities. |
| To construct telemedicine systems and health room informatization interconnection in Middle and West China. | 0.67 billion dollars | Telemedicine networks covering all China were primarily established. About 81.3% of provinces/ cities had set up province-level population health information platforms by 2016, and over 800 medical institutions in over 20 provinces/cities carried out telemedicine services; over 2200 county-level medical institutions and third-class hospitals in 1330 counties had set up telemedicine systems. |
| To support the construction of grass-rooted medical and health management information systems, and the construction of basic drug centralized purchasing and use information systems, and grass- rooted medical and health institution management information systems. | 0.75 billion dollars | Longitudinal and transversal medical informatization networks were primarily constructed. Grass-rooted medical and health institution comprehensive management information systems were used in 69% of counties (cities, districts) of China. Regional public health information platforms were set up in 453 counties (districts) from 113 prefecture-level cities in 14 provinces, and regional medical and health information systems interconnection and interworking were achieved to varying degrees. EHR coverage rate of Chinese residents was up to 76.4% by the end of 2016, and in the developed coast areas (e.g. Shanghai), the rate already exceeded 97% in 2013. |
| To support health card release to urban residents, application of environmental construction, registration and management of environmental construction, and establishment of health information sharing mechanism. | 0.1 billion dollars | Popularization of resident health cards was largely promoted throughout China. Health card release to residents, application of environmental construction, registration and management of environmental construction, and establishment of health information sharing mechanisms were especially supported in 15 provinces/cities. |
| To support the information exchange between 44 regional medical center hospitals in 31 provinces/cities and the National Health Commission Data Center. | 0.1 billion dollars | Construction of state-level medical information exchange platforms was promoted. Interconnection and interworking of the national platform with 31 provinces (regions, cities), Xinjiang Production & Construction Corps, and 44 hospitals of the State Council were realized, and relevant works of summarization, reporting and statistical analysis of data were started. |

Note:

The 24 provinces/cities in Middle and West China include Hebei, Anhui, Hainan, Shaanxi, Shanxi, Jiangxi, Chongqing, Gansu, Inner Mongolia, Henan, Sichuan, Qinghai, Liaoning, Hubei, Guizhou, Ningxia, Jilin, Hunan, Yunnan, Heilongjiang, Guangxi, Tibet, Xinjiang, and Xinjiang Production and Construction Corps.

The 16 cities selected for public hospital pilot reform include Shanghai, Shenzhen, Xiamen, Zhenjiang, Weifang, Luoyang, Wuhu, Ma'anshan, Zhuzhou, Ezhou, Kunming, Zunyi, Baoji, Xining, Anshan, and Qitaihe.

The 15 provinces/cities selected for resident health card pilot application include Liaoning, Henan, Hebei, Shaanxi, Sichuan, Hunan, Yunnan, Hubei, Chongqing, Anhui, Guizhou, Hubei, Jilin, Heilongjiang, and Fujian
